# Antibacterial activity of a *Tribolium castaneum* defensin in an *in vitro* infection model of *Streptococcus pneumoniae*

**DOI:** 10.1080/21505594.2019.1685150

**Published:** 2019-11-02

**Authors:** Nora S. Lindhauer, Wilhelm Bertrams, Anne Pöppel, Christina E. Herkt, Andre Wesener, Kerstin Hoffmann, Brandon Greene, Mark Van Der Linden, Andreas Vilcinskas, Kerstin Seidel, Bernd Schmeck

**Affiliations:** a Institute for Lung Research, Universities of Giessen and Marburg Lung Center, Philipps-University Marburg, Member of the German Center for Lung Research (DZL), Marburg, Germany; b Department of Bioresources, Fraunhofer Institute for Molecular Biology and Applied Ecology, Giessen, Germany; c Institute of Medical Bioinformatics and Biostatistics, Universities of Giessen and Marburg, Philipps-University Marburg, Marburg, Germany; d German National Reference Center for Streptococci, Department of Medical Microbiology, University Hospital RWTH Aachen, Aachen, Germany; e Institute for Insect Biotechnology, Justus-Liebig-University, Giessen, Germany; f Department of Medicine, Pulmonary and Critical Care Medicine, University Medical Center Giessen and Marburg, Philipps-University, Member of the German Center for Lung Research (DZL), Marburg, Germany

**Keywords:** Antimicrobial peptides, *Streptococcus pneumoniae*, macrophages, inflammation, insect, antibiotic resistance, defensin

## Abstract

*Streptococcus pneumoniae* (*S. pneumoniae*) is the most common bacterial cause of community-acquired pneumonia. Increasing rates of antibiotic-resistant *S. pneumoniae* strains impair therapy and necessitate alternative treatment options. In this study, we analysed insect-derived antimicrobial peptides (AMPs) for antibacterial effects on *S. pneumoniae* in a human *in vitro* infection model.

AMP effects on bacterial growth were examined by colony forming unit (CFU)-assays, and growth curve measurements. Furthermore, cytotoxicity to primary human macrophages was detected by measuring lactate-dehydrogenase release to the supernatant. One AMP (Defensin 1) was tested in a model of primary human monocyte-derived macrophages infected with *S. pneumoniae* strain D39 and a multi-resistant clinical isolate. Inflammatory reactions were characterised by qPCR and multiplex-ELISA.

In total, the antibacterial effects of 23 AMPs were characterized. Only *Tribolium castaneum* Defensin 1 showed significant antibacterial effects against *S. pneumoniae* strain D39 and a multi-resistant clinical isolate. During *in vitro* infection of primary human macrophages with *S. pneumoniae* D39, Defensin 1 displayed strong antibacterial effects, and consequently reduced bacteria-induced cytokine expression and release.

In summary, *Tribolium castaneum* Defensin 1 showed profound antibacterial effectivity against *Streptococcus pneumoniae* D39 and a multi-resistant clinical isolate without unwanted cytotoxic or inflammatory side effects on human blood-derived macrophages.

## Introduction

Approximately three million people die of pneumococcal infections worldwide every year [1], representing a high socio-economic burden of pneumococcal infection on society. *Streptococcus pneumoniae* (*S.p*.) is the most common cause of community-acquired pneumonia, affecting especially the young and the elderly [2]. Besides, it causes meningitis, otitis media, and other respiratory diseases. Penicillin resistance rates of pneumococci have increased up to 25% in Europe and hinder effective antibiotic treatment [3]. Worldwide, 20–30% of pneumococcal infections are caused by multidrug-resistant bacteria (i.e. bacteria resistant to more than three classes of antibiotics), mandating the development of alternative antibiotics [4].

Antimicrobial peptides (AMPs) are ancient effector molecules produced by the innate immune system of eukaryotic organisms to counteract viruses, bacteria, fungi, and parasites. The highest diversity of AMPs has been reported from insects [5]. Insect-derived AMPs can be classified according to their activities or their structural characteristics. In a structural context, three major classes are established: 1) AMPs that contain unusually high numbers of specific amino acid residues like proline or glycin (e.g. Apidaecin, Drosocin, Lebocin, Metchnikowin, Formaecin, Pyrrhocoricin, Metalnikowin); 2) linear α-helical AMPs peptides without cysteine residues such as cecropins, sarcotoxins, and stomoxyns; and 3) AMPs with a β-sheet globular structure stabilized by intramolecular disulphide bridges such as insect defensins[6].

Cationic AMPs are a subcategory of AMPs that are rich in cationic and hydrophobic residues, giving them an overall net positive charge[7]. In contrast, bacterial membranes are negatively charged and contain hydrophilic head groups in the outer leaflet, making them a suitable attack vector for AMPs[8]. This electrostatic interaction is one reason, amongst others, for AMP-driven pore formation in the bacterial envelope[9]. Additionally, some AMPs interact with intracellular targets[6], induce host immune cell chemotaxis[10], stimulate angiogenesis or promote wound healing[11].

In this study, we selected 23 AMPs based on their origin from different insects and their classification into different functional and structural classes (Table S1), and analyzed them for bactericidal activity against *S. pneumoniae* D39 and a multi-resistant clinical isolate. Only Defensin 1 from the model beetle *Tribolium castaneum* (Defensin 1 Genbank accession number: XM_968482[12]) was efficient against pneumococci. In addition, it reduced D39 survival and, consequently, bacteria-induced cytokine release in an infection model of primary human macrophages, but did not cause increased host cell cytotoxicity, hemolysis, inflammation or immunosuppression.

## Materials and methods

### Antimicrobial peptides

The tested insect-derived AMPs (Table S1) were produced by solid-phase synthesis as previously described (commissioned work by Panatecs GmbH, Tübingen Germany)[13]. Confirmation of correct formation of disulfide linkages in synthetic *Tribolium* Defensin 1 was done by mass spectrometric analysis of the fragment spectra of disulfide like tryptic peptides (data not shown). The insect AMPs were extracted to 80% purity by reverse-phase chromatography by GenScript (NJ, USA). Synthetic *Tribolium* Defensin 1 was resynthesized and processed to more than 95% purity.

### *S. pneumoniae* culture and growth kinetics

*S. pneumoniae* D39 – serotype 2 and a multi-resistant clinical isolate serotype 19A (AMR, resistant against Penicillin 8 µg/ml, Cefotaxim 4µg/ml, Erythromycin 256 µg/ml, Clindamycin 256 µg/ml, Tetracycline 32 µg/ml) were cultured as described before[14]. They were grown on sheep blood agar plates for 10 h and then transferred to Todd Hewitt Yeast (THY) medium (Carl Roth GmbH, Karlsruhe) at a density of 4 × 10^7^ bacteria/ml (Ultraspec 10 Cell Densitometer, Amersham Biosciences). Upon reaching a density of 2 × 10^8^ bacteria/ml, the bacterial suspension was diluted to 5 × 10^6^ bacteria/ml. To establish the optimal concentration ranges of AMP activity, 23 AMPs were diluted to 25 µM, 12.5 µM, 6.25 µM, 3.125 µM, and 1.56 µM and were added to the bacterial culture for further 10 h, or bacteria were left untreated. Additionally, Defensin 1 was incubated with bacteria for 24 h. The optical density of the culture was then measured automatically at 30-min intervals at OD_600nm_ with a TECAN Infinite M200 Pro plate reader for the indicated period.

### CFU

The absolute bacterial number of *S.p*. after treatment with Defensin 1 was quantified by colony forming unit count (CFU). Therefore, *S.p*. were grown as described above to 5 × 10^6^ bacteria/ml. After subsequent incubation for 5 h and 30 min in THY medium, AMPs were added at 12.5 µM, 6.25 µM, 3.125 µM or bacteria were left untreated. These AMP concentrations had been established by growth kinetic analyzes to have mild (3.125 µM), intermediate (6.25 µM) and strong (12.5 µM) inhibitory impact on bacterial replication. The treated bacteria were then cultivated for an additional 15 h at 37°C with 5% CO_2_ on sheep blood agar plates. Colonies were counted manually.

### Cell culture

Human monocytes were isolated from healthy donors by CD14 positive selection (CD14 Microbeads, Miltenyi Biotec). All donors gave informed written consent (Ethics approval number: 161/17). Monocytes were differentiated into blood-derived macrophages (BDMs) in the presence of 1% human AB-Serum. At day 6, differentiated cells were detached and seeded at the desired density in the presence of AB-Serum. After 24 h, cells were used for experiments.

### *S. pneumoniae* infection of BDMs

Infection of BDMs with *S. pneumoniae* strain D39 was carried out as described before[14]. Briefly, BDMs were infected at a multiplicity of infection (MOI) of 1 or 10. Control cells were left untreated. Either 1 or 5 h after infection, the pre-determined concentrations of Defensin 1 were added. 16 h after infection, RNA and supernatant were taken.

### LDH assay

Decreasing amounts of Defensin 1 (12.5 µM, 6.25 µM, 3.125 µM) were added to BDMs. After 24 h and 48 h, the supernatant was taken and lactate-dehydrogenase (LDH) release was measured as described before[14] with a TECAN Infinite M200 Pro plate reader to assess the cytotoxic impact of the AMP on BDMs.

### Hemolytic assay

Hemolytic assays were carried out using pig erythrocytes. Pig blood was obtained from a butcher and was mechanically treated to remove coagulants. Erythrocytes were harvested by centrifugation at room temperature for 5 min at 1500 g and were washed three times in phosphate-buffered saline (PBS). A suspension of the erythrocytes was prepared with a dilution factor of 1:5 in PBS. Serial dilutions of Defensin 1 (0.1, 1, 10, and 100 µM) were prepared and incubated with the erythrocyte suspension for 1 h at 37°C in a 96-well plate. Ensuing steps were carried out as previously described[15].

### RNA preparation and real time – PCR

For analysis of gene expression, total RNA isolation was carried out by phenol-chloroform extraction and RNA was reverse-transcribed to cDNA (High-Capacity RNA-to-cDNA kit, Thermo Fisher Scientific). Quantitative real-time PCR was performed with the following specific primer pairs:

IL-1β sense: 5ʹ-AGCTCGCCAGTGAAATGATGG-3ʹ; antisense: 5ʹ-CAGGTCCTGGAAGGAGCACTTC-3ʹ

IL-8 sense: 5ʹ-ACTGAGAGTGATTGAGAGTGGAC-3ʹ; antisense: 5ʹ-AACCCTCTGCACCCAGTTTTC-3ʹ

RPS18 sense: 5ʹ-GCGGCGGAAAATAGCCTTTG-3ʹ; antisense: 5ʹ-GATCACACGTCCACCTCATC-3ʹ

### Multiplex ELISA

The presence of cytokines in the BDM supernatant after infection and AMP treatment (6.25 µM) was assessed by Luminex Magpix Multiplex ELISA. BDM supernatants were prepared according to the manufacturer´s recommendations. The chosen cytokine panel included MIP1-a, MCP1, IL-1β, IL-6, IL-8, IL-10, IL-12p70, IL-23, LAP, and TNF-α.

### Statistics

Statistical interpretation of the multiplex ELISA required incorporation of data points below the detection limit and adjustment for the effect of multiple measurements made from the same replicates. We used the nonparametric-aligned ranks test (ART) of Hodges and Lehmann to compare cytokine secretion in treated and untreated samples within groups as well as in untreated samples between groups at 1 h and 5 h, respectively. In additional analyses, the same comparisons were made with pooled data across both time points. In these cases, data were stratified in the ART by replicates as well as time. All statistical analyses were performed using the R program for statistical computing.

All other statistical tests were performed as indicated in the figure legends. p-Values < 0.05 were considered significant.

## Results

### The AMP defensin 1 is effective against *S. pneumoniae* D39 and a multi-resistant clinical isolate

**Figure 1.**
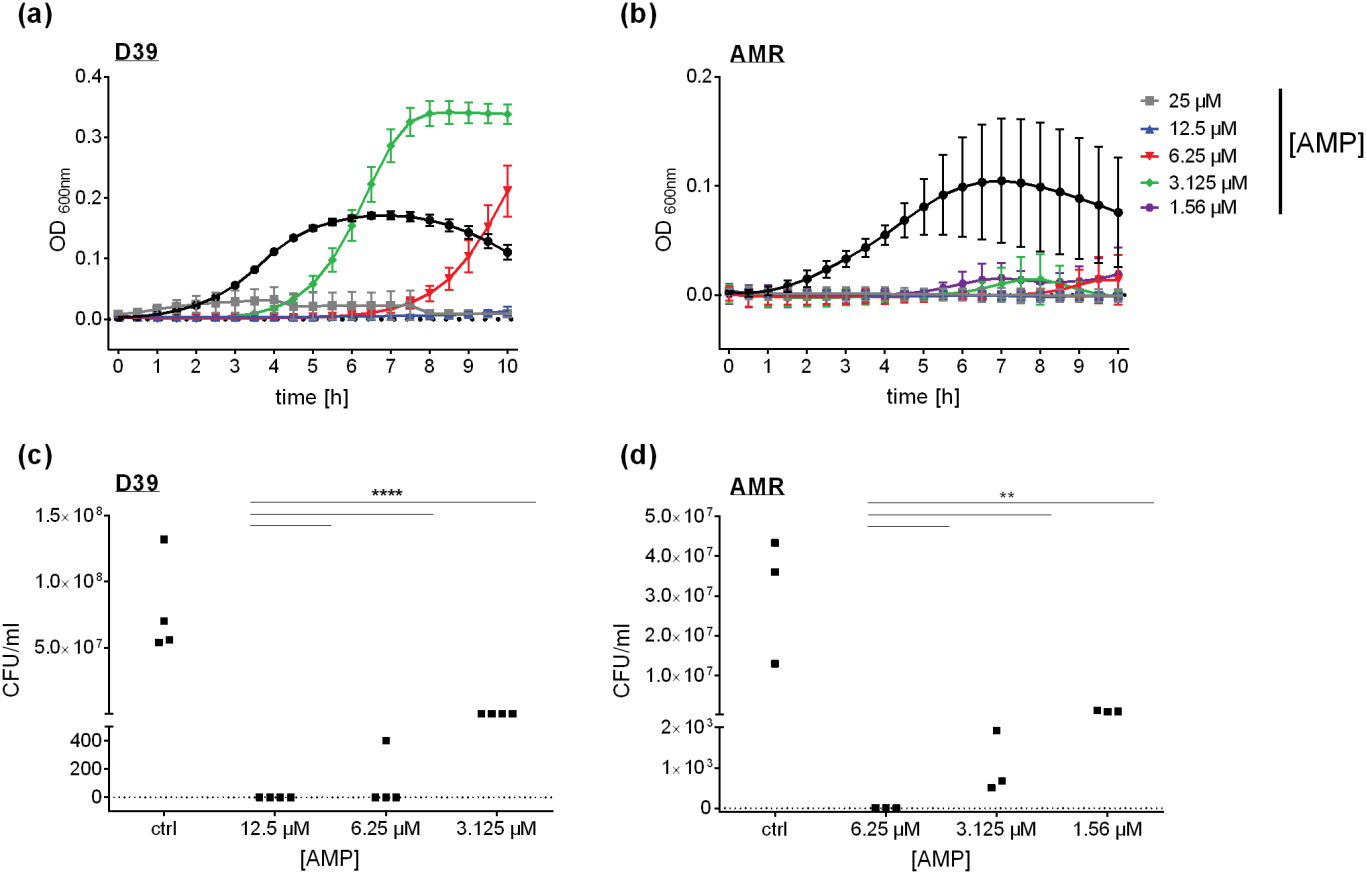
Defensin 1 significantly delays the growth of *S. pneumoniae* D39 and of multi-resistant *S. pneumoniae* as a function of concentration. *S.p*. were grown to an Optical Density (OD) 600nm of 0.4 in Todd Hewitt Yeast Medium. Upon dilution to OD_600nm_ = 0.01, *S.p*. D39 were incubated with declining amounts of Defensin 1 (25 µM, 12.5 µM, 6.25 µM, 3.125 µM) at 37°C with 5% CO_2_. OD_600nm_ was measured at 30 min intervals (a). The multi-resistant *S.p*. (AMR) were incubated with declining amounts of Defensin 1 (25 µM, 12.5 µM, 6.25 µM, 3.125 µM) under the same conditions (b). OD_600nm_ was measured at 30 min intervals. Bacterial growth of D39 (c) and the AMR strain (d) was monitored by colony forming unit assay. *S.p*. were grown and diluted as in (a) and (b). Statistical significance was assessed by two-way Anova, compared to corresponding control. **p < 0.01, ****p < 0.0001.

In search for antibacterial effectivity against *S. pneumoniae*, we selected 23 antimicrobial peptides from different insects (Table S1) and analyzed their bactericidal potential by monitoring growth kinetics. Only *Tribolium castaneum* Defensin 1 induced a significant reduction of *S. pneumoniae* growth at a minimal inhibitory concentration (MIC) of 12.5 µM (Figure 1(a) and S1). Of note, lower concentrations of Defensin 1 seemed to delay bacterial growth in THY medium, whereas higher concentrations prevented bacterial growth during the maximal time span of 24 h. To validate this observation, colony forming units (CFU) were counted after growth on sheep blood agar plates (Figure 1(c)). No bacteria could be detected after incubation with Defensin 1 with the MIC (12.5 µM). Upon treatment with lower concentrations (6.25 µM and 3.125 µM), significantly less bacteria survived compared to untreated pneumococci. The same approach was applied to a multi-resistant strain of *S. pneumoniae* (AMR). Here, 6.25 µM Defensin 1 considerably reduced bacterial growth (Figure 1(b)) and CFU count (Figure 1(d)). Lower concentrations (3.125 µM and 1.56 µM) still reduced bacterial growth in the CFU-Assay but showed less impact on the growth kinetics in liquid culture.

### Defensin 1 reduces *S. pneumoniae* survival and thereby inflammatory activation of macrophages

**Figure 2.**
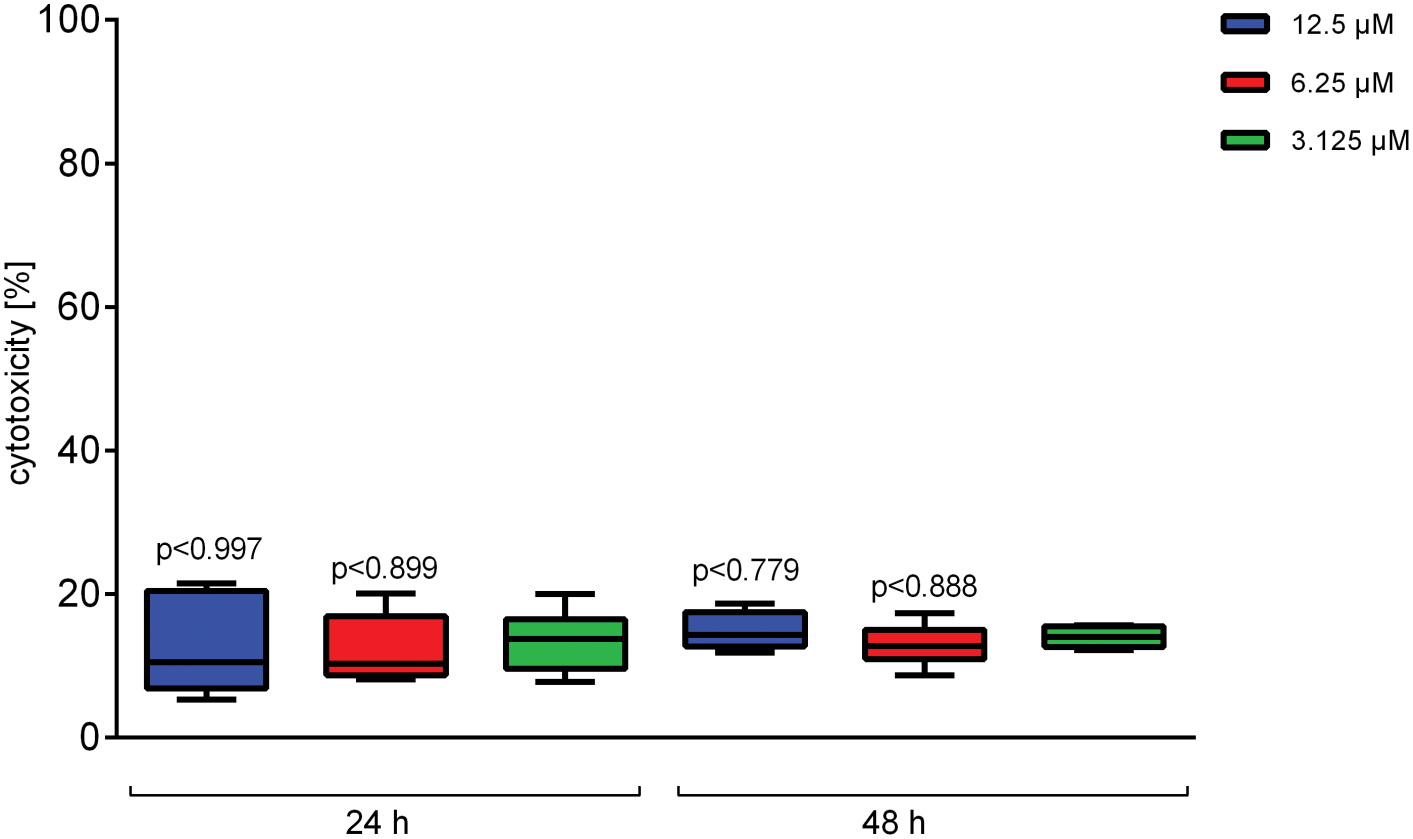
Defensin 1 has low cytotoxic impact on blood derived macrophages. After incubation of BDM with specified concentrations of Defensin 1 for either 24 or 48 hours, cytotoxicity was measured by LDH-Assay. Values were calculated based on 100% total lysis. Statistical significance was assessed by one-way Anova vs. lowest dose of AMP.

**Figure 3.**
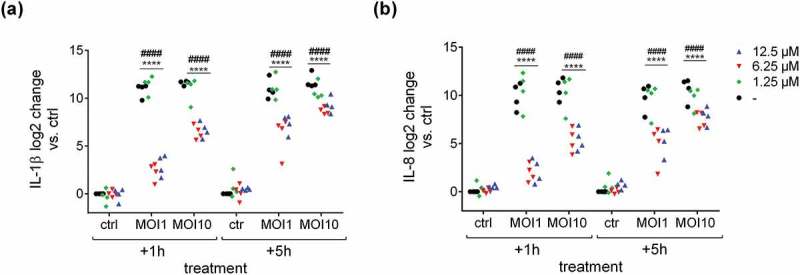
Defensin 1 leads to reduced cytokine mRNA expression in *S.p*. infected BDMs. 12.5 µM (MIC) and 6.25 µM of Defensin 1 lead to significantly less IL-1β mRNA (a) and IL-8 mRNA (b) expression for all MOIs and timepoints. 1.25 µM AMP showed no differences in interleukin expression compared to control (-). Log2 transformed data are shown. Statistical significance was assessed by two-way Anova, ^####^p < 0.0001 (12.5 µM vs. corresponding control), ****p < 0.0001 (6.25 µM vs. corresponding control.).

**Figure 4.**
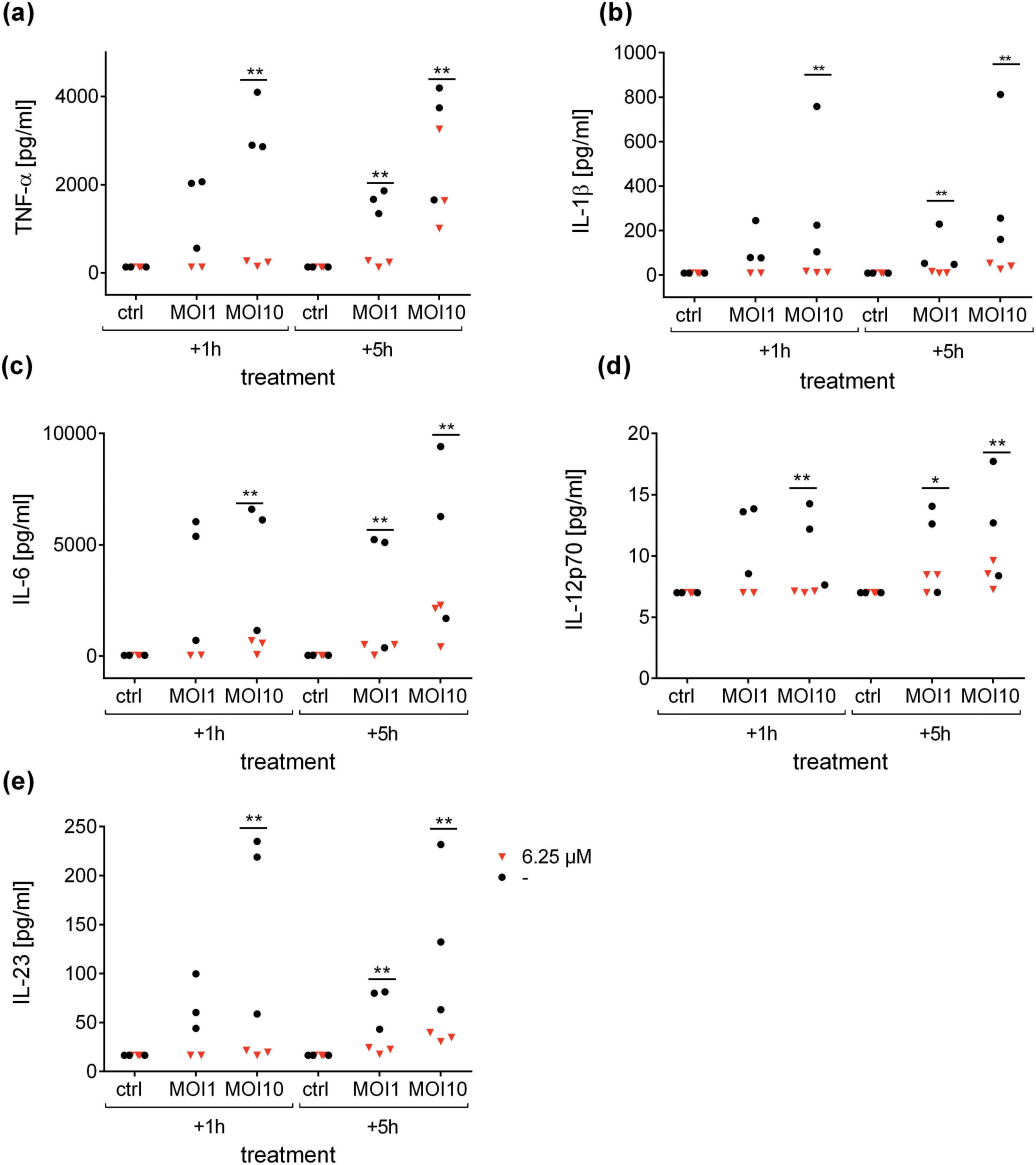
Defensin 1 reduces cytokine release from infected human macrophages. Supernatants from samples in Figure 3 were analyzed by Multiplex ELISA for different cytokines. Secretion levels of TNF-α (a), IL-1β (b), IL-6 (c), IL-12p70 (d) and IL-23 (e) after treatment with 6.25 µM Defensin 1 for 1 h or 5 h post infection, respectively, or left untreated for control (-) are shown. Statistical significance was assessed as described in the methods section, **p < 0.01, *p < 0.05 vs. corresponding control.

To elucidate the effect of Defensin 1 in an infection model, we determined AMP-induced cytotoxicity and inflammation in an *in vitro* model with BDMs. In preparation, hemolytic activity of Defensin 1 was determined to be minimal up to a concentration of 100 µM (Figure S2). The cytotoxic effect of Defensin 1 on BDMs was determined by LDH release which remained constant across the AMP concentration range and over time (Figure 2). AMP treatment was started 1 or 5 h after infection of BDM with *S. pneumoniae*. Defensin 1 concentrations of 6.25 µM and 12.5 µM had similar effects on mRNA expression of IL-1β and IL-8 after infection (MOI 1 and 10) for both time-points, measured by qPCR, whereas 1.25 µM had no effect in any condition (Figure 3). Therefore, we chose 6.25 µM Defensin 1 for the following experiment, since this dose also markedly delayed *S. pneumoniae* growth and only showed mild cytotoxicity. Defensin 1 administration to macrophages 1 h and 5 h after infection with D39 (MOI10) led to a reduction of bacteria-induced release of key inflammatory cytokines such as IL-1β, IL-6, and TNF-α (Figure 4). Sterile activation of BDMs by TNF-α was not altered significantly by Defensin 1 administration as shown by transcript levels of IL-8 and IL-1β (Fig S3).

## Discussion

*S. pneumoniae* is a major cause of pneumonia, meningitis, sepsis, bacteremia, and otitis media, and has developed increased resistance against multiple classes of antibiotics, including beta-lactams, macrolides, lincosamides, and trimethoprim-sulfamethoxazole[16]. This development mandates a global effort to search for natural compounds that could be used as new antimicrobials. Insect AMPs have recently emerged as a potential new treatment option for bacterial infection. Prior work documents the antibacterial effect of AMPs against the Gram-positive bacterium *Staphylococcus aureus*[17], as well as multidrug-resistant Gram-negative bacteria *Escherichia coli* and *Enterococcus faecalis*[18]. In this study, we selected 23 insect AMPs from three different classes, namely cysteine-rich peptides (e.g. Defensin 1), α-helical peptides (e.g. Cecropin, Sarcotoxin, Stomoxyn) and proline-rich peptides (e.g. Apidaecin, Drosocin, Lebocin, Metchnikowin, Formaecin, Pyrrhocoricin, Metalnikowin) and analyzed them for antibacterial activity against *S. pneumoniae* D39 and a multi-resistant clinical isolate. These particular peptides were chosen due to their ability to be synthetically produced and their characterization in previous studies [19–21].

Only Defensin 1 turned out to be effective against both strains. While a high dose of Defensin 1 completely abolished bacterial growth, lower concentrations led to a delay of growth which was recovered after 10 h of incubation. Defensin 1 represents one of three defensins identified in the genome of the model beetle *Tribolium castaneum* which are induced and released within the hemolymph upon activiation of innate immune responses[12]. The structural and functional analysis of *Tribolium* defensins revealed that they are primarily active against Gram-positive bacteria[5]. Supporting our findings, Defensin 1 also displays activity against Gram-positive multidrug-resistant *Staphylococcus aureus*[13].

Defensins can incapacitate or kill Gram-positive and Gram-negative bacteria[22] and have been described to attach to bacterial membranes, following proposed models such as barrel-stave, carpet, toroidal-pore and distorted toroidal pore [23,24], leading to loss of bacterial structural integrity. Defensin 1 possesses multiple disulfide bonds, which confer protection against degradation. This might also explain why Defensin 1 was the only effective AMP in our panel of 23 candidates. Another feature of Defensin 1 is the organization in beta-sheets. Alpha-helical peptides, such as cecropins, have been described to undergo conformational changes upon contact with membranes[8], while Defensin 1 does not. This might also be a determining factor for the antibacterial activity of Defensin 1.

Until now, the majority of AMP studies against *Streptococcus pneumoniae* has focused on mouse models or bacterial cultures[25]. We choose an *in vitro* infection model with human macrophages to maintain species concordance, and selected two treatment schedules: In a clinical environment, antimicrobial intervention is in most scenarios initiated after infection. We therefore investigated Defensin 1 activity in an early (1 h) and delayed (5 h) pneumococcal infection setting with human blood-derived macrophages to approximate the onset of an antimicrobial therapy in the clinic. Treatment starts 1 h after infection simulated a selective therapy, commonly used as a post-operative prevention of bacterial infections. The 5 h’ time point simulated post-infection therapy, as it is common in primary care. Some antibacterial treatments have been described to act intrinsically immune-modulatory (e.g., macrolides[26]), others have been found to activate the immune system in a deleterious way by destroying the bacteria (e.g. the Jarisch-Herxheimer Reaction[27]). Therefore, we analyzed the effects of Defensin 1 on the immune response. S. *pneumoniae* is sensed by the innate immune systems via various extracellular (TLR2, TLR4) and intracellular (NLRP3 inflammasome, TLR9, NOD2) receptors, leading to proteolytic cleavage of pro-IL-1β and to NFκB-mediated expression of other pro-inflammatory cytokines like, IL-8, TNF-α, IL-6, IL-12, and IL-23 [28,29]. Transcript expression of IL-1β and IL-8 were reduced upon Defensin 1 administration and concomitant infection. Therefore, we tested secretion of cytokines in response to infection with and without AMP treatment. The administration of Defensin 1 to *S. pneumoniae* infected BDMs significantly reduced secretion of TNF-α, IL-1β, IL-6, IL-12p70, and IL-23 in a time- and dose-dependent manner. As Defensin 1 was able to limit *S. pneumoniae* growth, and as it had no direct influence on cytokine secretion by itself, we hypothesize that Defensin 1 limits the pro-inflammatory activation of macrophages by efficient bacterial killing. Macrophages that were exposed to *S. pneumoniae* D39 for 5 h before Defensin 1 treatment also benefit from the treatment with Defensin 1. At MOI 1, there is only a trend to reduced cytokine release by Defensin 1, presumably because of the lower initial cytokine induction. Therefore, we attribute the reduced cytokine secretion to the lower bacterial load upon AMP treatment.

Cytokines that were also measured but which showed no significant change in secretion upon AMP treatment included IL-10, Latency-Associated-Peptide (LAP, a TGF-β precursor), MIP-1 α, MCP1, and IL-8. While IL-10 and LAP are predominantly of anti-inflammatory capacity, MIP-1 α, MCP1, and IL-8 are pro-inflammatory chemokines. It has been documented that AMPs can enhance chemokine production, e.g. MCP1, while inhibiting production of other cytokines[30]. We rule out such a pro- or anti-inflammatory effect of Defensin 1, since induction of IL-8 and IL-1β are not affected by AMP administration in the context of sterile TNF-α stimulation. Of note, there are substances that combine immunomodulation and bactericidal properties, such as peptide 1018, which has also been shown to be efficient against bacteria in biofilms[30].

It would be interesting to combine Defensin 1 with other factors, as previous work has documented cases of a cooperative effect of AMPs[31]. The combination of AMPs with different mechanisms[32] of action can potentiate their antibacterial effect, as it has been shown for the AMPs Abaecin plus Hymenoptaecin[31]. Additionally, the first findings indicate potentiating effects to be a general mechanism and not being restricted to peptides that are co-expressed in the same species. Of note, boosting the effects of AMPs in combination with conventional antibiotics is also conceivable [13,32] and could be used to reduce the required antibiotic dose or to sensitize resistant strains against traditional antibiotics. This also applies to putative synergistic effects of Defensin 1 and other AMPs. Additionally, investigation of the effect of Defensin 1 on infected lung epithelium is of further interest to gain a broader overview of the reaction of the human body to this particular molecule.

In summary, we found that Defensin 1 significantly reduced the growth of *Streptococcus pneumoniae* D39 and of a multi-resistant clinical isolate, showed low cytotoxic impact on human blood-derived macrophages and significantly reduced the cytokine secretion of TNF-α, IL-1β, IL-6, IL-12p70, and IL-23 while not displaying any immune-modulatory effects. Therefore, Defensin 1 represents a promising lead for the development of therapeutic compounds against *Streptococcus*-mediated pneumonia.
