# Supplementary figures and images for: Antibacterial activity of a Tribolium castaneum defensin in an in vitro infection model of Streptococcus pneumoniae
Source: Virulence. 2019 Nov 2;10(1):902–9. doi: 10.1080/21505594.2019.1685150 (PMC6844301; doi:10.1080/21505594.2019.1685150)

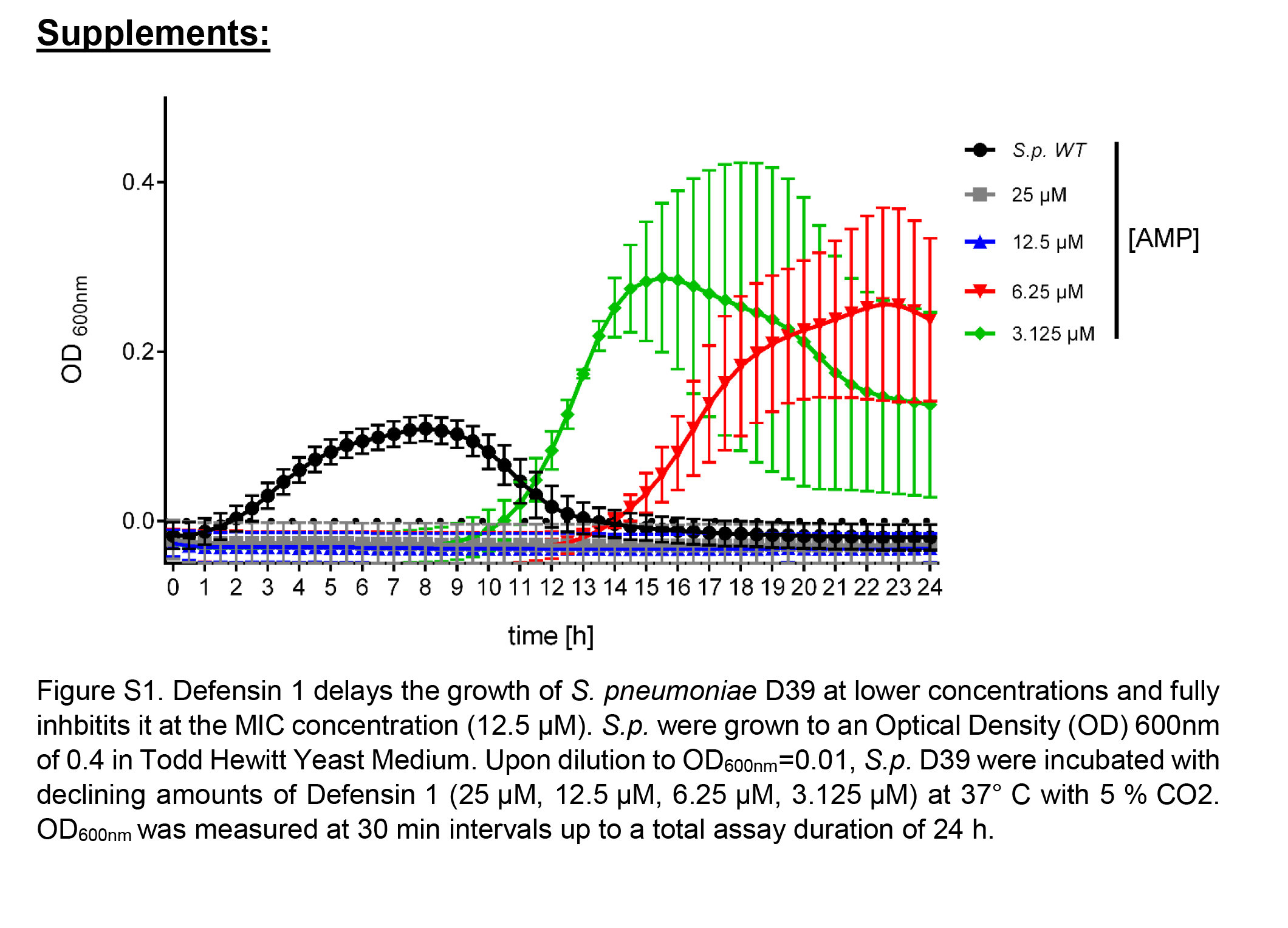

Supplement: Supplemental Material [file kvir-10-01-1685150-s001.zip › FigS1_Legend_final.tif]

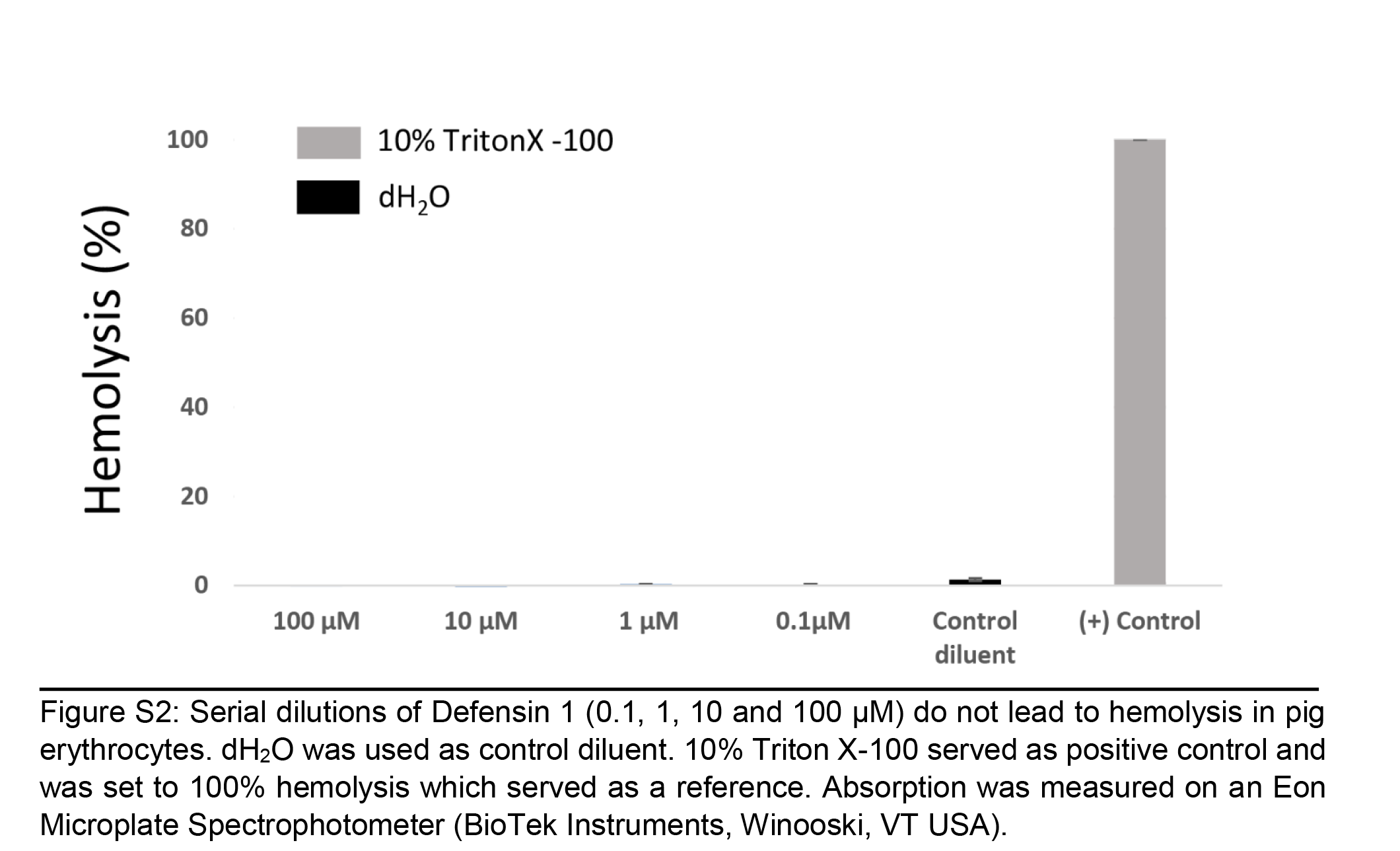

Supplement: Supplemental Material [file kvir-10-01-1685150-s001.zip › FigS2_legend_final.tif]

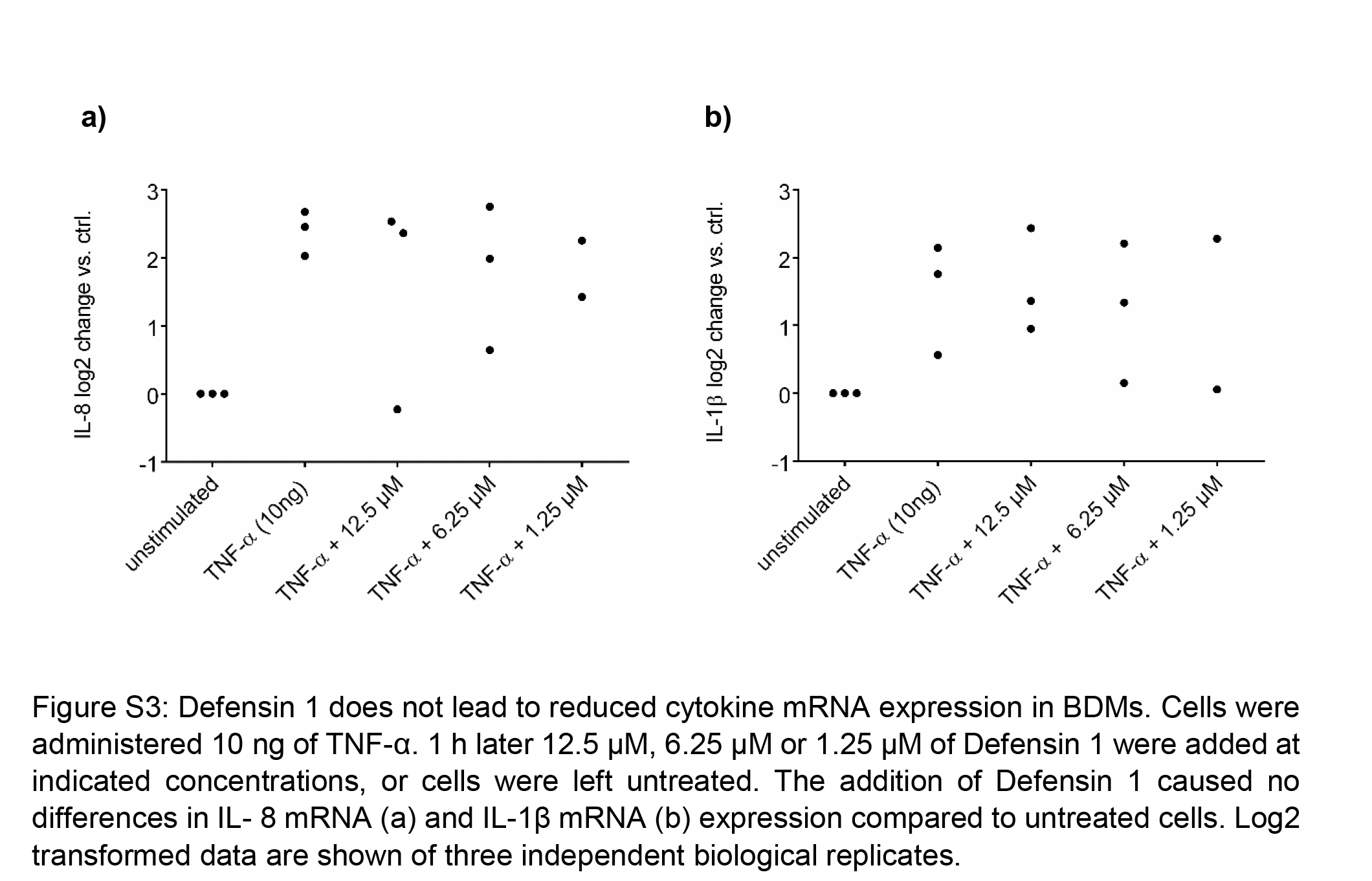

Supplement: Supplemental Material [file kvir-10-01-1685150-s001.zip › FigS3_legend_final.tif]

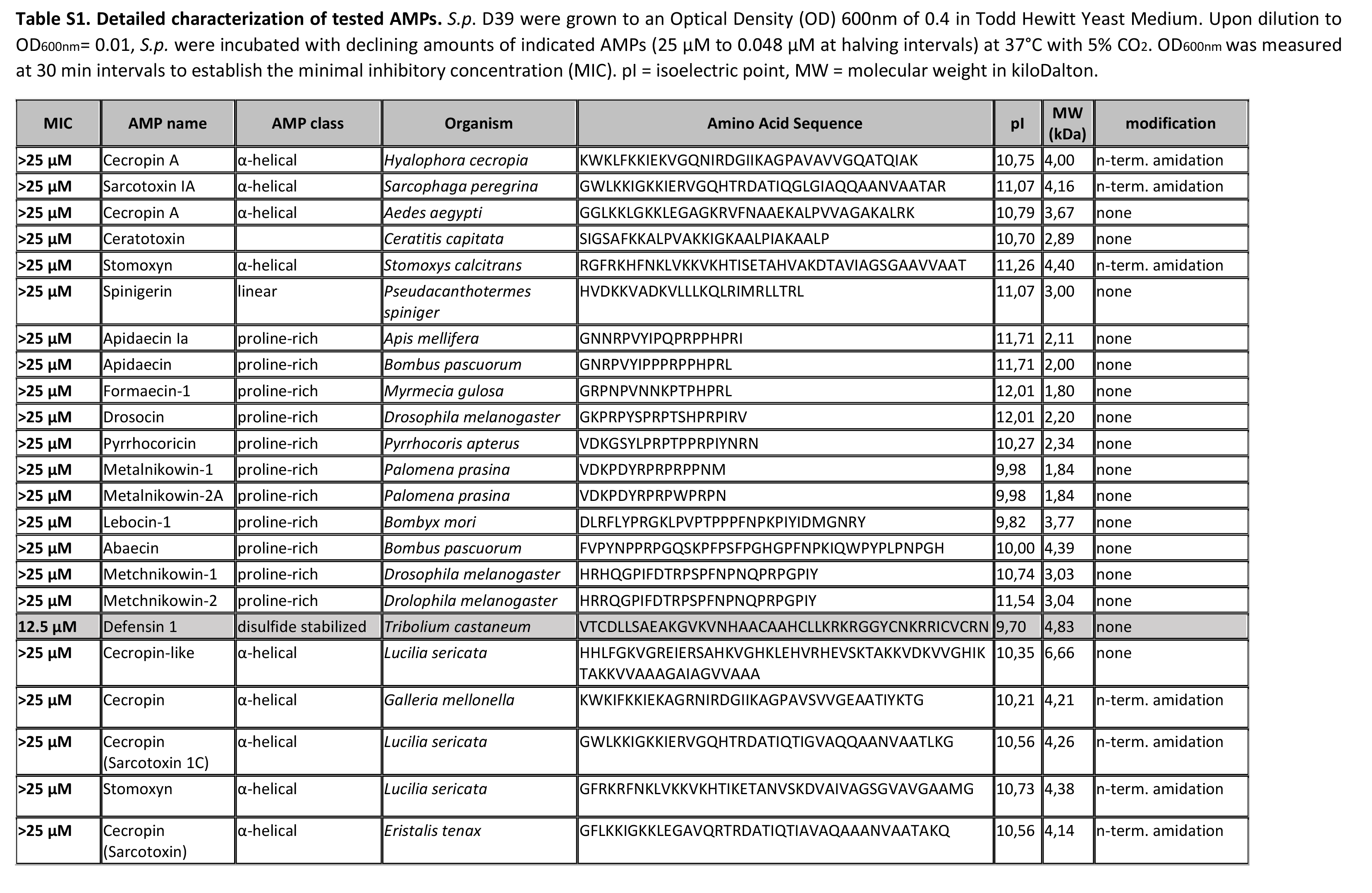

Supplement: Supplemental Material [file kvir-10-01-1685150-s001.zip › TableS1_final.tif]
